# Late-onset symptomatic hyperprolactinemia in 6-pyruvoyl-tetrahydropterin synthase deficiency

**DOI:** 10.1186/s13023-023-02963-4

**Published:** 2023-11-10

**Authors:** Rai-Hseng Hsu, Ni-Chung Lee, Hui-An Chen, Wuh-Liang Hwu, Tung-Ming Chang, Yin-Hsiu Chien

**Affiliations:** 1 Department of Pediatrics, National Taiwan University Hospital, Taipei, Taiwan; 2 Department of Medical Genetics, National Taiwan University Hospital, 8 Chung-Shan South Road, Taipei, 10041 Taiwan; 3 Department of Pediatrics, National Taiwan University College of Medicine, Taipei, Taiwan; 4 Center for Precision Medicine, China Medical University Hospital, Taichung, Taiwan; 5 Department of Pediatric Neurology, Changhua Christian Children’s Hospital, Changhua, Taiwan; 6 Department of Biological Science and Technology, College of Biological Science and Technology, National Yang Ming Chiao Tung University, Hsinchu, Taiwan

**Keywords:** Tetrahydrobiopterin deficiency, BH_4_, 6-Pyruvoyl-tetrahydropterin synthase deficiency, PTPS, Hyperphenylalaninemia, Hyperprolactinemia

## Abstract

**Background:**

Tetrahydrobiopterin (BH_4_) deficiency caused by 6-pyruvoyl-tetrahydropterin synthase (PTPS) deficiency is a rare disorder that is one of the major causes of hyperphenylalaninemia in Taiwan.

**Methods:**

In this study, we reviewed the clinical courses of 12 adolescent and adult patients (7 females and 5 males) with PTPS deficiency.

**Results:**

The patients were treated shortly after diagnosis through newborn screening with a combination of BH_4_, levodopa/carbidopa, and 5-OH-tryptophan. Their plasma phenylalanine and tyrosine levels were well controlled, and their prolactin levels were also decreased after treatment. However, their prolactin levels gradually rose as they grew into puberty, and at a current age of 27.5 [interquartile range (IQR 7.9)] years, five of the 12 patients had either highly elevated prolactin levels (> 100 ng/mL in one male patient, normal reference values, male < 11 ng/mL, female < 17 ng/mL) or symptoms, including irregular menstruation, amenorrhea, and breast swelling (in four female patients). The dosage of levodopa in these five patients (14.3 (IQR 3.0) mg/kg/day) was slightly higher than that in the other patients (*p* = 0.05). Magnetic resonance imaging studies did not reveal an increase in the size of the anterior pituitary gland, although a Rathke cleft cyst was found in one patient. Two patients received cabergoline treatment, which promptly lowered prolactin levels and relieved symptoms.

**Conclusions:**

Hyperprolactinemia is common in female patients with PTPS deficiency, especially after puberty. A long-acting dopamine agonist, such as cabergoline, may be a necessary adjunctive treatment for most patients with BH_4_ deficiency.

## Background

Hyperphenylalaninemia (HPA) is caused by variants either within the gene for the hepatic enzyme phenylalanine hydroxylase (PAH) or in genes involved in the production or recycling of tetrahydrobiopterin (BH_4_). According to the International Database of Tetrahydrobiopterin Deficiencies (BIODEFdb) [1], the most common cause of BH_4_ deficiency is 6-pyruvoyl-tetrahydropterin synthase (PTPS) deficiency (65% by summary in October 2015), followed by deficiencies of dihydropteridine reductase (DHPR, 25%), GTP cyclohydrolase I (GTPCH, 3%), and pterin-4α-carbinolamine dehydratase (PCD, 3%).

BH_4_ is a cofactor of PAH, tyrosine hydroxylase (TH), and tryptophan hydroxylase (TPH). Defects in BH_4_ biosynthesis or regeneration cause impaired synthesis of dopamine and serotonin, and furthermore the products of dopamine including adrenaline and noradrenaline, leading to motor dysfunction and even early death [2, 3]. Cerebrospinal fluid (CSF) concentrations of homovanillic acid (HVA) and 5-hydroxyindoleacetic acid (5-HIAA), the metabolites of dopamine and serotonin, are biomarkers of BH_4_ deficiency [4]. Pituitary prolactin secretion is under tonic and predominant inhibitory control exerted by hypothalamic dopamine secretion [5]. Serum prolactin levels rise in response to dopamine deficiency [2]. Serum prolactin levels are usually a part of the diagnostic work-up for BH_4_ deficiency [6–9]. In patients with PTPS deficiency, prolactin levels inversely correlate with growth [10]. However, the value of prolactin as a treatment monitoring biomarker has not yet been established.

Recently, prolactinoma as a cause of persistent hyperprolactinemia in PTPS deficiency was reported, and three adult patients with PTPS deficiency presented with persistent hyperprolactinemia even under high-dose levodopa therapy [11]. Their MRI scans revealed pituitary microadenoma. In another case report, a young man with DHPR deficiency developed movement disorders, seizures, and high prolactin levels despite levodopa supplementation, and a brain MRI study also suggested a pituitary microadenoma [4].

In Taiwan, BH_4_ deficiency accounts for approximately 30% of all cases of HPA, in contrast to 1% in Caucasian populations [12, 13]. Due to newborn screening, patients were treated with BH_4_, levodopa, and 5-hydroxytryptophan shortly after birth [12]. We observed symptomatic hyperprolactinemia in four of the seven female patients, even though none of them had pituitary microadenoma.

## Methods

Medical records of patients with PTPS deficiency who were regularly treated at National Taiwan University Hospital from January 1, 1991, to June 30, 2023, were reviewed. Patients were diagnosed through newborn screening for PKU due to hyperphenylalaninemia, followed by a BH_4_ loading test, urinary pterins, and mutation analysis. After diagnosis, patients were treated with BH_4_ (sapropterin dihydrochloride), levodopa/benserazide hydrochloride, and 5-HTP, and plasma amino acid analysis and prolactin level measurements were performed every 3 months. Data collected included recent body weight, body height, laboratory profile (phenylalanine, tyrosine, and prolactin), imaging studies, and medications. For patients with hyperprolactinemia, brain MRI with gadolinium enhancement was performed, and the depth of the pituitary gland was measured from sagittal sections at the midline. Serum prolactin levels were measured by radioimmunoassay. Amino acids were separated by an Intrada Amino Acid column (50 mm × 3 mm, Imtakt Corp, Japan) and analyzed by an API 4000 LC‒MS/MS system (Sciex Applied Biosystems). This retrospective chart review study was approved by the Institutional Review Board of the National Taiwan University Hospital, and the need for written informed consent was waived. For statistics, numbers are expressed as the median and interquartile range (IQR). Comparisons between two groups were performed by the Wilcoxon rank sum test.

## Results

A total of 13 patients with a diagnosis of PTPS deficiency after newborn screening were treated at National Taiwan University Hospital. Except for one male patient aged 5.4 years, the other 12 patients (7 females and 5 males) were older than 12 years (Table 1). The median age of these two adolescents and 10 adults was 27.5 (IQR 7.9) years. Pathogenic biallelic variants of the *PTS* gene were identified in all patients. All patients had normal growth, although two patients were overweight, with body mass index (BMI) values of 26.6 and 30.3. Some of the patients had borderline intellectual functioning, but this parameter was not included in the current study.

**Table 1:** Data from 12 adolescent and adult patients with PTPS deficiency

No	Sex	Age (years)	Phe^a^ (µM)	Tyr^a^ (µM)	Mean prolactin (ng/mL)	Peak prolactin (ng/mL)	Genotype	Sapropterin dihydrochloride^b^	Levodopa^b^	5-HTP^b^
**1**	**M**	**31.0**	**76.9**	**111**	**143.6**	**375.0**	**c.155G > A ho**	**2.8**	**11.2**	**0.0**
**2**	**F**	**20.7**	**60.8**	**91.4**	**54.7**	**89.4**	**c.155G > A ho**	**6.3**	**14.8**	**4.2**
3	F	27.9	67.6	81.2	31.1	66.4	c.155G > A ho	3.0	6.0	3.0
4	F	27.5	96.7	78.2	8.5	37.8	c.259C > T, c.286G > A com he	1.3	8.9	3.8
**5**	**F**	**27.5**	**71.7**	**76.2**	**73.1**	**195.7**	**c.155G > A ho**	**2.0**	**11.8**	**2.9**
7	M	26.6	100.0	98.4	22.5	87.1	c.73C > G, c.259C > T com he	2.2	9.0	4.5
8	M	30.5	73.0	89.6	31.6	69.4	c.155G > A ho	3.3	13.1	4.9
**9**^**c**^	**F**	**14.5**	**122.0**	**189.0**	**73.2**	**321.4**	**c.155G > A, c.314 + 1G > A** com he	**4.4**	**17.6**	**8.8**
**10**^**c**^	**F**	**27.9**	**60.6**	**134.0**	**127.2**	**302.1**	**c.155G > A ho**	**4.1**	**14.3**	**6.1**
11	M	14.2	87.9	93.5	4.9	59.7	c.259C > T, c.331G > A com he	4.5	7.5	2.9
12	F	20.6	114.0	284.0	11.8	42.7	c.259C > T, c.286G > A com he	5.3	13.2	6.6
13^**d**^	M	31.4	N.A	N.A	N.A	22.5	c.155G > A ho	N.A	N.A	N.A
Median		27.5	76.9	93.5				3.3	11.8	4.4
IQR		7.9	28.7	37.1				2.0	4.8	2.6

*N.A.* not available, *IQR* interquartile range, *Ho* homozygous, *com he* compound heterozygous

^a^Latest level

^b^mg/kg/day

^c^Dostinex 0.5 mg/wk

^d^No recent data

Bold indicates the five patients with either highly elevated prolactin levels or symptoms

**Fig. 1:**
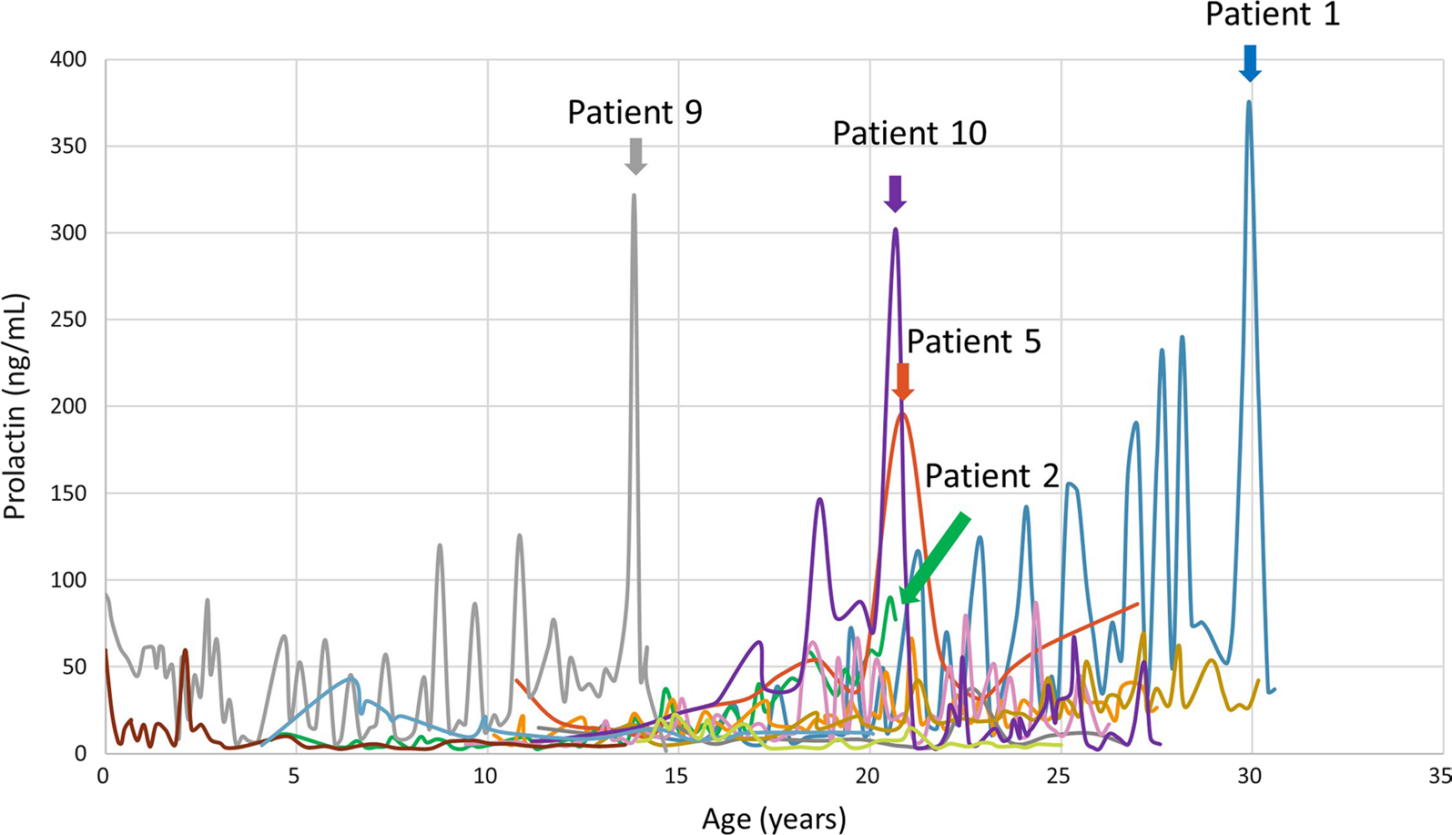
Prolactin levels of the 12 patients with PTPS deficiency. Patients 1, 2, 5, 9, and 10 had significant or symptomatic elevation of prolactin. Normal reference values of prolactin: male < 11 ng/mL, female < 17 ng/mL

**Fig. 2:**
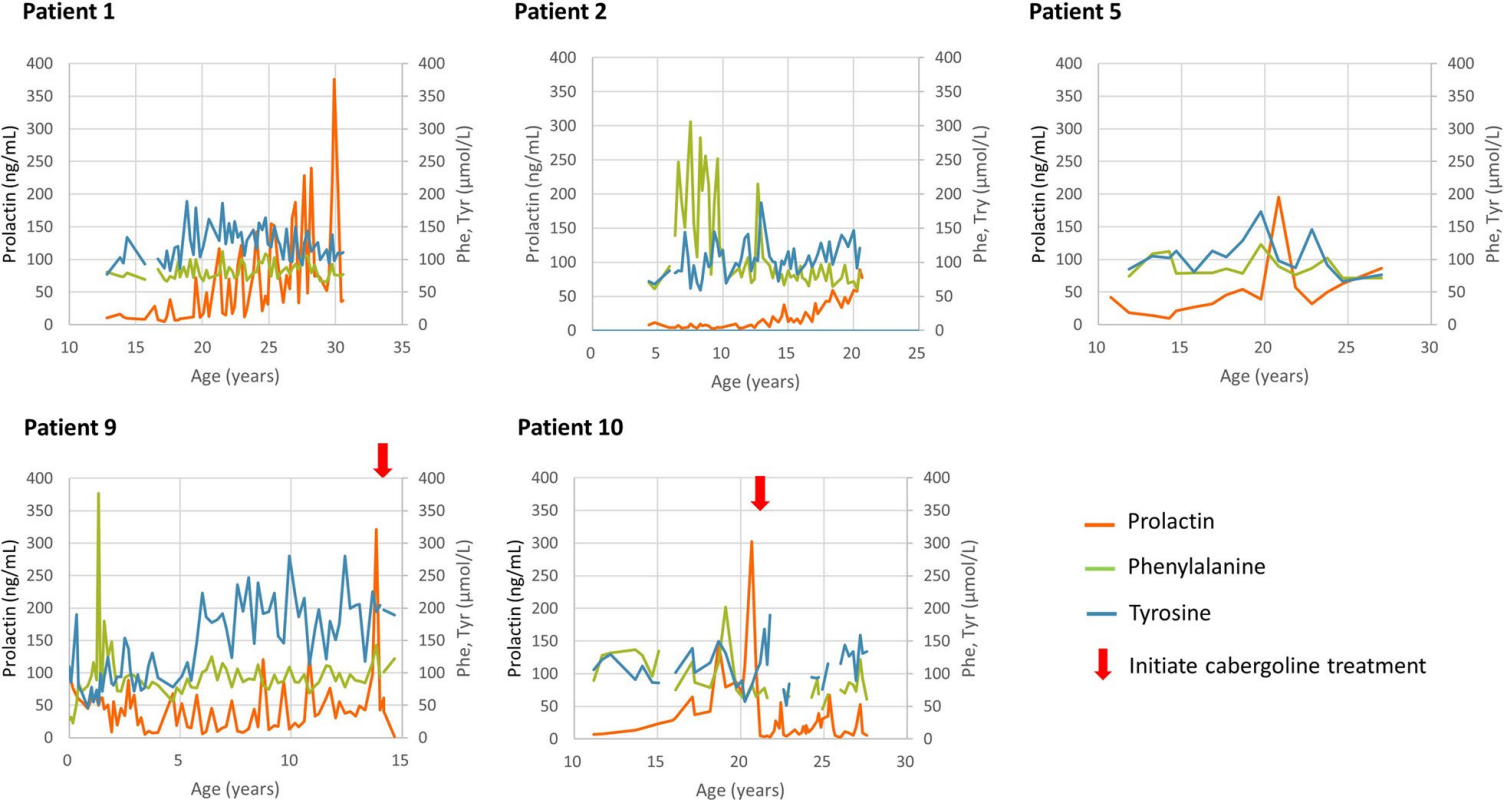
Timeline of prolactin, phenylalanine, and tyrosine levels of Patients 1, 2, 5, 9, and 10. Cabergoline was administered to Patients 9 and 10, with the initiation time denoted with red arrows

**Fig. 3:**
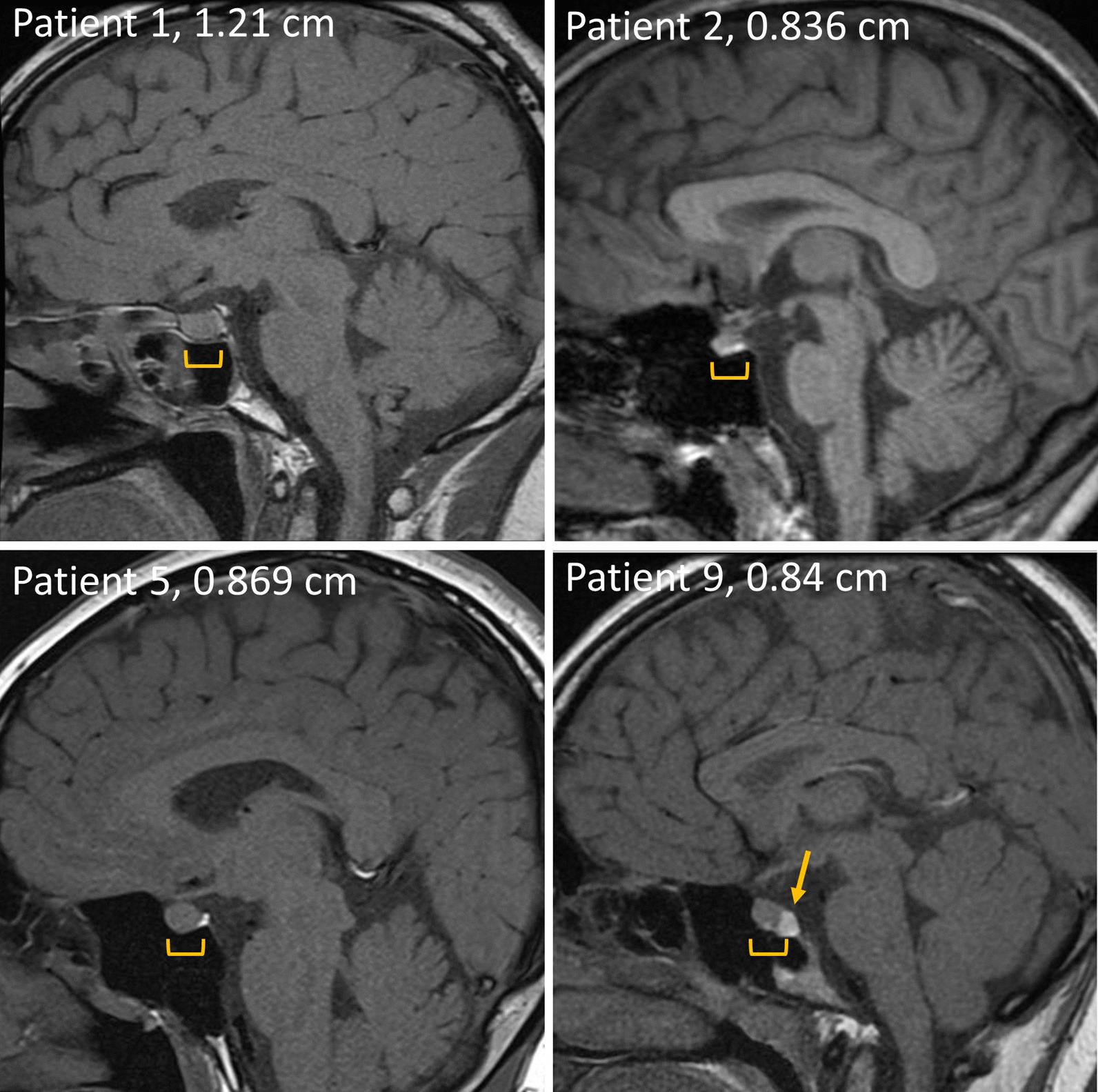
Brain MRI sagittal section images at the midline of Patients 1, 2, 5, and 9. The depth of the anterior pituitary gland (bracket) is marked on the images. A pituitary Rathke cleft cyst was found in Patient 9 (arrow)

The patients were treated immediately after diagnosis. Their initial responses to treatment were adequate, with low phenylalanine and prolactin levels. Because there was only one child in this series and he was followed for a relatively shorter period than the other adolescent and adult patients, the following analyses excluded this child, and the total number of patients was 12. Their most recent mean dosage of BH_4_ was 3.3 (IQR 1.5) mg/kg/day, and the dosage of levodopa was 11.8 (IQR 4.8) mg/kg/day in 3–4 divided doses. Their most recent phenylalanine level was 76.9 (IQR 28.7) µmol/L, and their tyrosine level was 93.5 (IQR 37.1) µmol/L. The control of phenylalanine levels was largely attributed to oral BH_4_ medication. Patient 4 experienced akinesia, especially in the morning, at the age 22. Her symptom improved after an increase of levodopa dosage, and her prolactin level also decreased from 37.8 ng/mL to normal. Patient 3 has experienced symptoms suggestive of an overdose of levodopa. However, her CSF neurotransmitter metabolite levels were normal, and her prolactin levels remained stable after a decrease of levodopa dosage. When patients reached adolescence, drug compliance tended to fluctuate, and prolactin levels rose when doses were missed (Fig. 1). However, prolactin levels increased (normal reference values, male < 11 ng/mL, female < 17 ng/mL) persistently in five patients (Patients 1, 2, 5, 9, and 10) despite good drug compliance (Fig. 2). The median dosage of levodopa in these five patients was 14.3 (IQR 3.0) mg/kg/day, slightly higher than that in the remaining six patients (9.0 (IQR 4.2) mg/kg/day, recent data were not available for Patient 13) (*p* = 0.0519). Four of the five patients were females, and they all had symptoms including irregular menstruation and breast swelling. Two patients were treated with cabergoline (Dostinex®) 0.5 mg per week. MRI studies in four patients revealed an anterior pituitary gland depth of 0.836–1.21 mm (Fig. 3). Compared with the reported mean pituitary depths of 10.59 ± 1.71 mm and 10.49 ± 1.57 mm in males and females, respectively [14], our patients did not show remarkable enlargement of the pituitary gland. A Rathke cleft cyst was found in Patient 9 (Fig. 3, arrow).

Patient 1 was a 31-year-old man. He maintained stable phenylalanine and tyrosine levels throughout his life. However, serum prolactin levels rose in his 20s despite an increased dose of levodopa and an increased frequency from 3 to 4 times per day. Brain MRI suggested a pituitary microadenoma, but there was no enlargement of the anterior pituitary gland. Patient 2 was a 20-year-old woman with normal phenylalanine levels but increased prolactin levels with periodic breast swelling since adolescence. Brain MRI revealed no obvious lesions. Patient 5 was a 27-year-old woman who had highly elevated prolactin levels and irregular menstruation at the age of 20 years. Patient 9 was a 14-year-old girl who also maintained normal phenylalanine levels throughout her life. A substantial increase of prolactin level up to 321.38 ng/mL was noted at the age of 13. Her menstruation was irregular, and breast swelling was also reported. Her brain MRI showed a pituitary Rathke cleft cyst. After the initiation of cabergoline treatment for 5 months, the prolactin levels returned to a normal range. Patient 10 was a 29-year-old woman with a gradual increase in prolactin levels since her teens. Brain MRI at the age of 7 showed no pituitary lesion, but follow-up MRI was refused. Cabergoline was prescribed in 2016, at age 21, because of amenorrhea, and the treatment promptly lowered prolactin levels; she now had regular menstruation, and there was no more breast swelling.

## Discussion

In the current study, we demonstrated that a significant portion (42%) of adolescent or adult patients with BH_4_ deficiency suffered from hyperprolactinemia, and in female patients, the condition was aggravated by the menstrual cycle, which resulted in symptoms including irregular menstruation, amenorrhea, and painful breast swelling. In contrast to a previous report [4, 11], there was no evidence of prolactinoma in our patients. A Rathke cleft cyst, a benign, cystic remnant that develops between the two lobes of the pituitary gland, has been associated with prolactin secretion impairment [15], but it is more likely an incidental finding in our study.

Prolactin is part of the diagnostic work-up in BH_4_ deficiency and an indirect marker for adequate L-DOPA treatment [9]. Prolactin is synthesized mainly by the anterior pituitary gland, and pituitary prolactin secretion is under tonic and predominant inhibitory control exerted by hypothalamic dopamine secretion [5]. The best-known physiological stimulus for prolactin release is nursing, and during menstrual cycles, the estradiol signal triggers a surge of prolactin release. Because BH_4_ has poor blood‒brain barrier penetration, levodopa, which can be converted to dopamine by aromatic l-amino acid decarboxylase (AADC), is currently the standard treatment to replenish dopamine in BH_4_ deficiency. In our cases, after supplementation with BH_4_ and levodopa with/without 5-HTP, prolactin levels generally decreased well before puberty. However, even in the presence of a peripheral decarboxylase inhibitor, such as carbidopa or benserazide, the elimination half-life of levodopa is only approximately 1.5 h [16]. Conventional treatment with BH_4_, 5-hydroxytryptophan, and levodopa/carbidopa suppresses prolactin levels for merely a few hours [17]. Pramipexole, a D2 agonist with an elimination half-life of 5–10 h, has been used to treat BH_4_ deficiency, but impulse control disorders (gambling, compulsive buying, and hypersexuality) were observed in patients treated with a higher dose of pramipexole [18]; therefore, this long-acting formulation is not used.

Prolactinoma is the most common type of pituitary adenoma, accounting for approximately half of pituitary tumors [19]. Cabergoline, a long-lasting selective dopamine D2-receptor agonist, is often used to treat prolactinomas [20]. Cabergoline therapy for prolactinomas has been shown to normalize prolactin levels and induce tumor regression [21]. The advantage of cabergoline over other dopamine agonists in the treatment of prolactinomas is likely its very long elimination half-life of 60 h or more.

The etiology of the reoccurrence of hyperprolactinemia in our early-treated PTPS patients is unknown. Hypothalamic dopamine inhibits the secretion of prolactin in a complex way. It is likely that the dopamine levels caused by a few doses of oral levodopa are not sufficient for adequate suppression of prolactin secretion in patients with BH_4_ deficiency. However, prolactin did achieve normal levels using similar treatment regimens before puberty, implying that other etiologies, such as repeated fluctuation causing hyperstimulation of prolactin, should be considered. MRI did not show an enlarged gland, although we do not know if the cells are hyperactive. Another hypothesis is the daily interruption of levodopa, such as not taking levodopa at night for over 8–12 h. Although we have not compared the different regimens, such as taking levodopa for no more than 6 h and longer than 6 h, complaints such as amenorrhea suggest more persistent hyperprolactinemia in those patients. In addition, in female patients, prolactin secretion is regulated by the menstrual cycle, which potentially further exaggerates the inappropriate responses in our PTPS-deficient patients. However, because of the limitation of the retrospective chart review study design, we did not have information regarding the time of prolactin measurement, the menstrual cycle, or the time of daily levodopa supplementation. Further study will be needed to understand the exact mechanism of hyperprolactinemia in adult PTPS patients.

## Conclusions

We demonstrate that symptomatic hyperprolactinemia is a frequent finding in female patients with BH_4_ deficiency caused by PTPS deficiency. Adjunctive treatment with a long-acting dopamine agonist, cabergoline, should be considered, especially when symptoms occur. Determination of the long-term outcomes of hyperprolactinemia, with or without cabergoline treatment, will require further follow-up of these patients.

## Data Availability

The data that support the findings of this study are not openly available due to reasons of sensitivity and are available from the corresponding author upon reasonable request.
